# Ethnicity and risks of severe COVID‐19 outcomes associated with glucose‐lowering medications: A cohort study

**DOI:** 10.1111/dom.14872

**Published:** 2022-09-29

**Authors:** Francesco Zaccardi, Pui San Tan, Carol Coupland, Baiju R. Shah, Ash Kieran Clift, Defne Saatci, Martina Patone, Simon J. Griffin, Hajira Dambha‐Miller, Kamlesh Khunti, Julia Hippisley‐Cox

**Affiliations:** ^1^ Leicester Real World Evidence Unit, Leicester Diabetes Centre University of Leicester Leicester UK; ^2^ Nuffield Department of Primary Care Health Sciences University of Oxford Oxford UK; ^3^ Division of Primary Care, School of Medicine University of Nottingham Nottingham UK; ^4^ Department of Medicine, University of Toronto; Division of Endocrinology, Sunnybrook Health Sciences Centre Institute for Clinical Evaluative Sciences Toronto Ontario Canada; ^5^ Cancer Research UK Oxford Centre University of Oxford Oxford UK; ^6^ Primary Care Unit, School of Clinical Medicine University of Cambridge Cambridge UK; ^7^ MRC Epidemiology Unit, School of Clinical Medicine University of Cambridge Cambridge UK; ^8^ Primary Care Research Centre University of Southampton Southampton UK

## 1 INTRODUCTION

During the early phases of the COVID‐19 pandemic, diabetes became associated with a poorer prognosis,^1^ with an approximately three‐fold increased risk of a COVID‐19 death in those with diabetes compared with those without.^2^, ^3^ In an effort to understand this association, there was an increasing interest in the role of glucose‐lowering medications on the risk of COVID‐19 outcomes, given their pharmacological differences and potential direct effect on shared immunometabolic pathways^4^: the available evidence would suggest small absolute increased rates of COVID‐19 mortality with some dipeptidyl peptidase 4 inhibitors (DPP‐4i, insulin) and reduced with other medications [metformin (MF), sodium‐glucose cotransporter‐2 inhibitors (SGLT‐2i), sulphonylurea].^5^

Alongside the role of diabetes, multiple large observational studies also showed higher risks of COVID‐19‐related hospitalization, intensive care unit admission and death in people from ethnic minority populations.^6^, ^7^

As type 2 diabetes is more prevalent in ethnic minority populations—particularly South Asians—determining the risk of COVID‐19 outcomes in relation to different glucose‐lowering therapies has implications for both patients and health care professionals.^8^ We therefore designed a cohort study within the QResearch UK nationwide database to quantify the associations between prescriptions of glucose‐lowering medications and (a) COVID‐19 mortality, and (b) COVID‐19 hospitalization in different ethnic subgroups during the first wave of the pandemic.

## 2 METHODS

Details about population definition, exposure, outcomes and statistical analysis are reported in the Appendix S1.

## 3 RESULTS

Of the 624 771 people with type 2 diabetes, 56.1% were men and the mean ± SD age and diabetes duration at index date were 67.0 ± 13.4 and 9.7 ± 7.4 years, respectively; the mean body mass index was 30.5 ± 6.0 kg/m^2^ (Table 1). More than half of the included people were of white ethnicity (63.2%), followed by unknown ethnicity (13.2%), South Asian (11.7%), black (5.6%), Asian (3.3%) and other (3.1%). Diabetes duration was roughly similar across ethnic groups (mean varied between 8.8 and 9.8 years). The mean body mass index was slightly lower in South Asian (28.3 kg/m^2^) and Asian (27.6 kg/m^2^) compared with other ethnicities (between 30.0 and 31.1 kg/m^2^). Although the proportions varied, the three most common comorbidities across the ethnic groups were consistently hypertension, coronary heart disease and asthma.

**TABLE 1:** Baseline characteristics and COVID‐19 outcomes during follow‐up by ethnicity

	Asian	Black	South Asian	White	Other	Not recorded	All people
No. of people (%)	20 451 (3.3)	34 938 (5.6)	73 068 (11.7)	394 663 (63.2)	19 067 (3.1)	82 584 (13.2)	624 771 (100)
Age (years)	61.3 (12.9)	63.6 (13.5)	60.7 (13.6)	68.8 (12.9)	61.6 (13.1)	68.0 (13.4)	67.0 (13.4)
Diabetes duration (years)	9.1 (7.3)	9.7 (7.7)	9.8 (7.7)	9.8 (7.2)	8.8 (7.3)	9.6 (7.6)	9.7 (7.4)
Sex (men)	11 563 (56.5)	17 286 (49.5)	40 109 (54.9)	224 327 (56.8)	9999 (52.4)	47 175 (57.1)	350 459 (56.1)
HbA1c (mmol/mol)	56.8 (15.7)	59.0 (19.5)	58.1 (16.6)	55.9 (16.0)	58.6 (18.7)	56.4 (16.2)	56.5 (16.5)
Body mass index (kg/m^2^)	27.6 (4.8)	30.4 (5.8)	28.3 (5.1)	31.1 (6.1)	30.0 (5.8)	30.6 (6.0)	30.5 (6.0)
Non‐cancer disease							
Chronic obstructive pulmonary disease	434 (2.1)	701 (2.0)	1978 (2.7)	35 132 (8.9)	505 (2.6)	4917 (6.0)	43 667 (7.0)
Bronchiectasis	179 (0.9)	215 (0.6)	676 (0.9)	5225 (1.3)	133 (0.7)	883 (1.1)	7311 (1.2)
Asthma	2782 (13.6)	4158 (11.9)	11 496 (15.7)	65 016 (16.5)	2581 (13.5)	11 036 (13.4)	97 069 (15.5)
Hypersensitivity pneumonitis	–	–	46 (0.1)	381 (0.1)	–	73 (0.1)	524 (0.1)
Pulmonary hypertension	50 (0.2)	115 (0.3)	125 (0.2)	1466 (0.4)	71 (0.4)	218 (0.3)	2045 (0.3)
Pulmonary fibrosis	45 (0.2)	58 (0.2)	215 (0.3)	1452 (0.4)	35 (0.2)	230 (0.3)	2035 (0.3)
Hypertension	10 781 (52.7)	23 011 (65.9)	38 615 (52.8)	250 075 (63.4)	10 731 (56.3)	48 244 (58.4)	381 457 (61.1)
Coronary heart disease	2437 (11.9)	2471 (7.1)	11 945 (16.3)	70 030 (17.7)	1970 (10.3)	13 293 (16.1)	102 146 (16.3)
Stroke	830 (4.1)	2244 (6.4)	3839 (5.3)	35 009 (8.9)	989 (5.2)	6959 (8.4)	49 870 (8.0)
Atrial fibrillation or flutter	493 (2.4)	961 (2.8)	1660 (2.3)	41 080 (10.4)	611 (3.2)	7598 (9.2)	52 403 (8.4)
Congestive cardiac failure	512 (2.5)	1342 (3.8)	2712 (3.7)	25 333 (6.4)	662 (3.5)	4557 (5.5)	35 118 (5.6)
Venous thromboembolism	245 (1.2)	1285 (3.7)	1252 (1.7)	19 687 (5.0)	523 (2.7)	3781 (4.6)	26 773 (4.3)
Peripheral vascular disease	221 (1.1)	701 (2.0)	1179 (1.6)	18 077 (4.6)	321 (1.7)	3063 (3.7)	23 562 (3.8)
Dementia	374 (1.8)	1408 (4.0)	1719 (2.4)	15 666 (4.0)	511 (2.7)	3119 (3.8)	22 797 (3.6)
Parkinson disease	107 (0.5)	143 (0.4)	347 (0.5)	3001 (0.8)	72 (0.4)	611 (0.7)	4281 (0.7)
Epilepsy	141 (0.7)	351 (1.0)	671 (0.9)	7191 (1.8)	259 (1.4)	1310 (1.6)	9923 (1.6)
Cerebral palsy	–	–	27 (0.0)	298 (0.1)	–	48 (0.1)	406 (0.1)
Motor neurone disease	–	–	22 (0.0)	146 (0.0)	–	23 (0.0)	202 (0.0)
Huntington disease	–	–	–	48 (0.0)	–	–	67 (0.0)
Multiple sclerosis	9 (0.0)	42 (0.1)	35 (0.0)	1328 (0.3)	32 (0.2)	260 (0.3)	1706 (0.3)
Down syndrome	–	–	–	126 (0.0)	–	–	184 (0.0)
Fracture^a^	389 (1.9)	508 (1.5)	1702 (2.3)	25 886 (6.6)	364 (1.9)	4528 (5.5)	33 377 (5.3)
Cirrhosis	113 (0.6)	151 (0.4)	426 (0.6)	4173 (1.1)	113 (0.6)	753 (0.9)	5729 (0.9)
Cancer							
Myeloma	22 (0.1)	98 (0.3)	71 (0.1)	488 (0.1)	36 (0.2)	120 (0.1)	835 (0.1)
Leukaemia	31 (0.2)	63 (0.2)	133 (0.2)	1565 (0.4)	32 (0.2)	324 (0.4)	2148 (0.3)
Blood	130 (0.6)	296 (0.8)	499 (0.7)	4871 (1.2)	159 (0.8)	982 (1.2)	6937 (1.1)
Lung	25 (0.1)	39 (0.1)	101 (0.1)	1867 (0.5)	48 (0.3)	286 (0.3)	2366 (0.4)
Ovarian	22 (0.1)	38 (0.1)	66 (0.1)	664 (0.2)	18 (0.1)	118 (0.1)	926 (0.1)
Colorectal	100 (0.5)	248 (0.7)	291 (0.4)	5987 (1.5)	113 (0.6)	1103 (1.3)	7842 (1.3)
Renal	81 (0.4)	119 (0.3)	232 (0.3)	5024 (1.3)	86 (0.5)	927 (1.1)	6469 (1.0)
Liver	13 (0.1)	10 (0.0)	38 (0.1)	347 (0.1)	6 (0.0)	52 (0.1)	466 (0.1)
Oral	22 (0.1)	25 (0.1)	98 (0.1)	686 (0.2)	21 (0.1)	111 (0.1)	963 (0.2)
Pancreatic	11 (0.1)	24 (0.1)	18 (0.0)	460 (0.1)	12 (0.1)	91 (0.1)	616 (0.1)
Thyroid	22 (0.1)	20 (0.1)	73 (0.1)	350 (0.1)	32 (0.2)	61 (0.1)	558 (0.1)
Gastro‐oesophageal	20 (0.1)	53 (0.2)	61 (0.1)	822 (0.2)	13 (0.1)	173 (0.2)	1142 (0.2)
Breast	291 (1.4)	501 (1.4)	873 (1.2)	9726 (2.5)	270 (1.4)	1884 (2.3)	13 545 (2.2)
Cervical	14 (0.1)	31 (0.1)	23 (0.0)	552 (0.1)	9 (0.0)	98 (0.1)	727 (0.1)
Uterine	48 (0.2)	77 (0.2)	153 (0.2)	2016 (0.5)	50 (0.3)	415 (0.5)	2759 (0.4)
Prostate	162 (0.8)	1152 (3.3)	435 (0.6)	9163 (2.3)	337 (1.8)	1788 (2.2)	13 037 (2.1)
Glucose‐lowering medications							
Alpha‐glucosidase inhibitors	28 (0.1)	34 (0.1)	72 (0.1)	197 (0.0)	18 (0.1)	29 (0.0)	378 (0.1)
Dipeptidyl peptidase 4 inhibitors	4214 (20.6)	6721 (19.2)	16 733 (22.9)	68 471 (17.3)	3451 (18.1)	13 549 (16.4)	113 139 (18.1)
Thiazolidinediones	380 (1.9)	556 (1.6)	1878 (2.6)	8140 (2.1)	330 (1.7)	1814 (2.2)	13 098 (2.1)
Glucagon‐like peptide‐1 agonists	358 (1.8)	907 (2.6)	1621 (2.2)	18 429 (4.7)	644 (3.4)	3131 (3.8)	25 090 (4.0)
Meglitinides	102 (0.5)	131 (0.4)	317 (0.4)	586 (0.1)	59 (0.3)	95 (0.1)	1290 (0.2)
Sodium‐glucose cotransporter‐2 inhibitors	1989 (9.7)	2416 (6.9)	8008 (11.0)	39 778 (10.1)	1828 (9.6)	7928 (9.6)	61 947 (9.9)
Sulphonylureas	4773 (23.3)	7857 (22.5)	17 993 (24.6)	71 852 (18.2)	4186 (22.0)	13 931 (16.9)	120 592 (19.3)
Metformin	15 159 (74.1)	23 674 (67.8)	55 350 (75.8)	246 636 (62.5)	13 117 (68.8)	49 413 (59.8)	403 349 (64.6)
Insulin	1743 (8.5)	4690 (13.4)	7725 (10.6)	57 438 (14.6)	2231 (11.7)	10 935 (13.2)	84 762 (13.6)
Any glucose‐lowering medication	16 289 (79.6)	26 594 (76.1)	60 100 (82.3)	294 585 (74.6)	14 510 (76.1)	58 883 (71.3)	470 961 (75.4)
No. of glucose‐lowering medications	1.4 (1.1)	1.3 (1.1)	1.5 (1.1)	1.3 (1.1)	1.4 (1.1)	1.2 (1.1)	1.3 (1.1)
Outcomes							
COVID‐19 related death	75 (0.4)	316 (0.9)	320 (0.4)	2300 (0.6)	86 (0.5)	430 (0.5)	3527 (0.6)
COVID‐19‐related admission	200 (1.0)	630 (1.8)	743 (1.0)	3854 (1.0)	258 (1.4)	726 (0.9)	6411 (1.0)

*Note*: Shown are frequencies (%) for categorical variables and mean (SD) for continuous; rates are reported with 95% confidence intervals. If in a row there were cells with frequency ≤5, all row‐wise cells ≤20 were suppressed.

^a^ Hip, wrist, spine, humerus.

Abbreviations: HbA1c, glycated haemoglobin.

In the 3 month preceding the index date, 64.6% were prescribed MF, 19.3% sulphonylureas, 18.1% DPP‐4i, 13.6% insulin, 9.9% SGLT‐2i and ≤4% any of the other glucose‐lowering medications [alpha‐glucosidase inhibitors, thiazolidinediones (TZD), glucagon‐like peptide‐1 agonists (GLP‐1RA), meglitinides]; approximately 24.6% had no prescriptions of glucose‐lowering medications (Table 1). This pattern was generally consistent across ethnic groups.

During the study period, there were 3527 COVID‐19‐related deaths (0.6%, 7.5 per 1000 person‐years) and 6411 COVID‐19 related hospitalizations (1.0%, 13.6 per 1000 person‐years) (Table 1). COVID‐19 death and hospitalization rates showed a progressive increase with a peak at about 2‐3 months after the index date (March/April 2020) and a decline thereafter (Figure S1).

**FIGURE 1:**
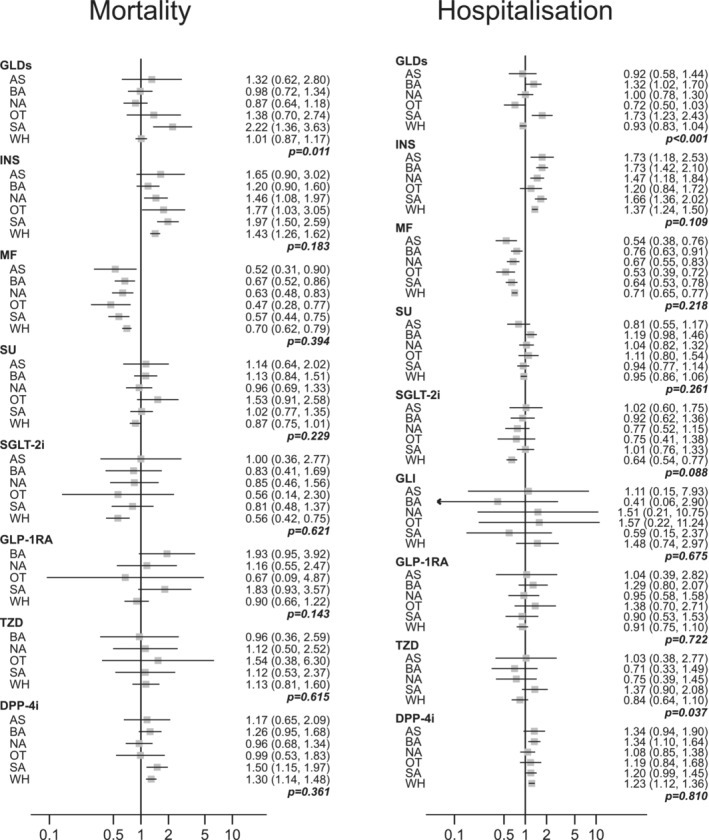
Adjusted hazard ratios for COVID‐19 death and hospitalization across ethnic groups. Adjusted hazard ratios comparing people with versus without the specific glucose‐lowering medication and *p*‐values for heterogeneity (interaction) across the ethnic groups. Because of imprecision in the estimates (few events), hazard ratios are not shown for mortality in Asian ethnic group for glucagon‐like peptide‐1 agonists (GLP‐1RA) and thiazolidinediones (TZD), and all ethnic groups for meglitinides and alpha‐glucosidase inhibitors; and for hospitalization in other ethnic group for TZD, and all ethnic groups for alpha‐glucosidase inhibitors. AS, Asian; BA, black; DPP‐4i, dipeptidyl peptidase 4 inhibitors; GLDs, any glucose‐lowering medication; GLI, meglitinides; INS, insulin; MF, metformin; NA, not reported; OT, other; SGLT‐2i, sodium‐glucose cotransporter‐2 inhibitors; SA, South Asian; SU, sulphonylureas; WH, white

Following adjustment for possible confounders, there was no evidence of different associations with COVID‐19 mortality rates between ethnic groups for most glucose‐lowering medications (Figure 1). Rates were, however, generally higher in people with versus without insulin [hazard ratios (HRs) from 1.20 (95% confidence interval: 0.90‐1.60) in to 1.97 (1.50‐2.59)], with no evidence of heterogeneity across ethnic groups (*p* = .183). Conversely, rates were consistently lower in people with versus without MF [HRs from 0.47 (0.28‐0.77) to 0.70 (0.62‐0.79)], with no evidence of heterogeneity (*p* = 0.394). Mortality rates were also lower in people of white ethnicity with versus without SGLT‐2i [HR 0.56 (0.42‐0.75); *p* = 0.621 for heterogeneity across ethnic groups] and higher in people of white [1.30 (1.14‐1.48)] or South Asian [1.50 (1.15‐1.97)] ethnicity with versus without DPP‐4i (*p* = 0.361 for heterogeneity; Figure 1). Evidence of statistically significant heterogeneity (*p* = 0.011) was observed only comparing any versus no glucose‐lowering therapy, with the highest HR for COVID‐19 mortality in South Asians [HR 2.22 (1.36‐3.63)] compared with all other ethnic groups.

Results for COVID‐19 hospitalization mirrored those for mortality (Figure 1). Rates were generally higher in people with versus without insulin [HRs from 1.20 (95% confidence interval: 0.84‐1.72) to 1.73 (1.18‐2.53)], with no evidence of heterogeneity across ethnic groups (*p* = .109); and consistently lower in people with versus without MF [from 0.53 (0.39‐0.72) to 0.76 (0.63‐0.91)], with no evidence of heterogeneity (*p* = .218). Hospitalization rates were also lower in people of white ethnicity with versus without SGLT‐2i [HR 0.64 (0.54‐0.77); *p* = .088 for heterogeneity across ethnic groups] and higher in people of white [1.23 (1.12‐1.36)], South Asian [1.20 (0.99‐1.45)], and black [1.34 (1.10, 1.64)] ethnicity with versus without DPP‐4i (*p* = .810 for heterogeneity; Figure 1). While the results suggested a higher excess in hospitalization rates associated with TZD prescription in South Asians compared with all other ethnic groups (*p* = 0.037 for heterogeneity), the excess in rates was more marked for any versus no glucose‐lowering therapy prescriptions in South Asians [1.73 (1.23‐2.43)] compared with other ethnic groups (*p* < 0.001 for heterogeneity).

## 4 CONCLUSIONS

Although poor glycaemic control has been linked to a higher risk of death in people with COVID‐19 and studies have reported several potential mechanisms through which DPP‐4i, SGLT‐2i, GLP‐1RA or MF may increase or lower the risk of COVID‐19 complications,^4^, ^9^, ^10^ current evidence does not suggest a large excess in the absolute risk of COVID‐19 mortality in relation to specific glucose‐lowering medications.^5^ Moreover, two randomized controlled trials in hospitalized patients with type 2 diabetes and COVID‐19 indicate no difference in clinical improvement comparing the DPP‐4i linagliptin to standard care,^11^ and in organ dysfunction or death comparing the SGLT‐2i dapagliflozin with placebo.^12^

Whether associations between glucose‐lowering drugs and COVID‐19 outcomes differed across ethnic groups, however, was unknown. Our results indicate a higher relative risk of COVID‐19 mortality and hospitalization associated with the prescription of insulin—possible related to an increased inflammatory milieu in patients with type 2 diabetes and COVID‐19 on insulin—and a lower risk of these outcomes in people prescribed MF, with no statistical difference in the magnitude of the associations across ethnic groups.^4^ Moreover, for both outcomes SGLT‐2i prescription was associated with a lower risk in white people and DPP‐4i with a higher risk in white people and South Asians. Our findings can in part be compared with a previous UK study using different data, as investigators assumed associations with glucose‐lowering medications of the same magnitude across ethnicity. Notwithstanding, in people with type 2 diabetes a higher risk of COVID‐19‐related mortality was observed with a prescription of insulin and a lower risk with MF and SGLT‐2i,^5^ in line with our findings.

To our knowledge, this is the first study exploring ethnicity‐specific associations between glucose‐lowering medications and COVID‐19 outcomes. The main strength is the large population‐based sample with adjustment for several potential confounders, including comorbidities, duration of diabetes and glycaemic control (glycated haemoglobin). However, as with all observational studies, causality cannot be inferred: residual confounding for unmeasured confounders remains possible; we could not account for potential differences in kidney function, although it is unlikely that adding information on the estimated glomerular filtration rate would materially change the magnitude/direction of associations given the several confounders accounted for and the possible association of some of them with renal function (i.e. diabetes duration). Furthermore, treatment indication could have biased our results, particularly in view of the presence of heterogeneity across ethnic groups when comparing any versus no prescription. Our data related to the first pandemic wave in the UK; since then, the majority of the UK population has received a vaccine and different coronavirus variants emerged, with effects on the risk of infection transmission and its progression to disease, severe disease and death.^13^ We considered as exposure a prescription issued within 3 months before the index date; however, a prescription does not necessarily denote dispensation while adherence to therapy may vary among different ethnic groups.^14^ Lastly, the definition of the outcomes was based on the clinical codes as reported on the death certificates and hospital records; it may be possible that the presence of other concomitant clinical conditions have affected the decision to admit a patient or have contributed to death (“dying with vs. due to COVID‐19”).

Personalized decisions among the optimal strategy to reduce glucose levels should also account for the possible side effects of the glucose‐lowering medications, as some could increase the risk of hypoglycaemia (i.e. sulphonylureas and insulin) while others are neutral in this respect (DPP‐4i, GLP‐1RA, MF, TZD and SGLT‐2i). Class‐specific effects have been reported in previous randomized controlled trials for GLP‐1RA (gastrointestinal),^15^, ^16^, ^17^ TZD (cardiovascular),^18^ and SGLT‐2i (genitourinary, metabolic)^17^, ^19^; however, if, and to what extent, these effects contribute to the heterogeneous risk of COVID‐19‐related hospitalization and mortality across the diverse glucose‐lowering medications has been little investigated.

In conclusion, our study confirms previous evidence about the potential for small absolute risk differences in COVID‐19 hospitalization and death across glucose‐lowering medications. However, for each medication, no clear differences were observed among ethnic groups, with the greater excess risk of mortality and hospitalization in South Asians with a prescription of any glucose‐lowering therapy possibly related to residual confounding. Further studies could help estimate the absolute risks in relation to the varying rates of SARS‐CoV‐2 infection and COVID‐19 outcomes.

## AUTHOR CONTRIBUTIONS

All authors contributed to development of research questions and formulated the study design; study conceptualisation was led by Kamlesh Khunti and Francesco Zaccardi.Francesco Zaccardi performed the statistical analyses. Clinical codes were developed and checked by Julia Hippisley‐Cox, Ash Kieran Clift, Pui San Tan, Francesco Zaccardi. Francesco Zaccardi, Pui San Tan, Kamlesh Khunti wrote the first draft of the manuscript, which was critically revised for important intellectual content by all other authors. All authors contributed to interpretation of results and approved the final version of the manuscript. The corresponding author attests that all listed authors meet authorship criteria and that no others meeting the criteria have been omitted. Julia Hippisley‐Cox is the guarantor.

## FUNDING INFORMATION

This study is jointly funded by UKRI and NIHR [COV0130 /MR/V027778/1]. Simon J. Griffin is supported by an MRC Epidemiology Unit programme: MC_UU_12015/4. The University of Cambridge has received salary support in respect of Simon J. Griffin from the NHS in the East of England through the Clinical Academic Reserve. Ash Kieran Clift is funded by a Clinical Research Fellowship from Cancer Research UK (C2195/A31310). Julia Hippisley‐Cox has received grants from the National Institute for Health Research, Oxford, John Fell Oxford University Press Research Fund, Cancer Research UK (grant number C5255/A18085) through the Cancer Research UK Oxford Centre, and the Oxford Wellcome Institutional Strategic Support Fund (204826/Z/16/Z) during the conduct of the study. Kamlesh Khunti and Francesco Zaccardi are supported by the National Institute for Health Research (NIHR) Applied Research Collaboration East Midlands (ARC EM) and the NIHR Leicester Biomedical Research Centre (BRC) (NIHR200171). Hajira Dambha‐Miller is a National Institute for Health Research funded Academic Clinical Lecturer.

## CONFLICT OF INTEREST

KK is a Member of the Scientific Advisory Group for Emergencies (SAGE), Director of the University of Leicester Centre for Black Minority Health and Trustee of the South Asian Health Foundation. PST reports past consultation with AstraZeneca and Duke‐NUS outside the submitted work. SJG has received honoraria from Astra Zeneca for contributing to postgraduate education meetings for primary care teams. JH‐C is a member of the Scientific Advisory Group for Emergencies (SAGE), the Independent Expert group for novel Covid therapeutics, and chair of the risk stratification subgroup of the NERVTAG. She is an unpaid director of QResearch, a not‐for‐profit organisation which is a partnership between the University of Oxford and EMIS Health who supply the QResearch database used for this work, and is a founder and shareholder of ClinRisk Ltd and was its medical director until 31 May 2019; ClinRisk produces open and closed source software to implement clinical risk algorithms (outside this work) into clinical computer systems. All other authors declare that they have no competing interests.

### PEER REVIEW

The peer review history for this article is available at https://publons.com/publon/10.1111/dom.14872.

## ETHICS STATEMENT

The QResearch project has been independently peer‐reviewed and received ethics approval by the QResearch Scientific board (REC 18/EM/0400; project reference OX102).

## Supporting information


**APPENDIX S1** Supporting Information

## Data Availability

To guarantee the confidentiality of personal and health information, only the authors have had access to the data during the study in accordance with the relevant license agreements. Access to QResearch data is according to the information on the QResearch website (www.qresearch.org).

## References

[1] Singh AK , Gillies CL , Singh R , et al. Prevalence of co‐morbidities and their association with mortality in patients with COVID‐19: a systematic review and meta‐analysis. Diabetes Obes Metab. 2020;22(10):1915‐1924. 32573903 10.1111/dom.14124 PMC7361304

[2] Barron E , Bakhai C , Kar P , et al. Associations of type 1 and type 2 diabetes with COVID‐19‐related mortality in England: a whole‐population study. Lancet Diabetes Endocrinol. 2020;8(10):813‐822. 32798472 10.1016/S2213-8587(20)30272-2 PMC7426088

[3] Wu J , Zhang J , Sun X , et al. Influence of diabetes mellitus on the severity and fatality of SARS‐CoV‐2 (COVID‐19) infection. Diabetes Obes Metab. 2020;22(10):1907‐1914. 32496012 10.1111/dom.14105 PMC7300679

[4] Drucker DJ . Diabetes, obesity, metabolism, and SARS‐CoV‐2 infection: the end of the beginning. Cell Metab. 2021;33(3):479‐498. 33529600 10.1016/j.cmet.2021.01.016 PMC7825982

[5] Khunti K , Knighton P , Zaccardi F , et al. Prescription of glucose‐lowering therapies and risk of COVID‐19 mortality in people with type 2 diabetes: a nationwide observational study in England. Lancet Diabetes Endocrinol. 2021;9(5):293‐303. 33798464 10.1016/S2213-8587(21)00050-4 PMC8009618

[6] Nafilyan V , Islam N , Mathur R , et al. Ethnic differences in COVID‐19 mortality during the first two waves of the coronavirus pandemic: a nationwide cohort study of 29 million adults in England. Eur J Epidemiol. 2021;36(6):605‐617. 34132940 10.1007/s10654-021-00765-1 PMC8206182

[7] Mathur R , Rentsch CT , Morton CE , et al. Ethnic differences in SARS‐CoV‐2 infection and COVID‐19‐related hospitalisation, intensive care unit admission, and death in 17 million adults in England: an observational cohort study using the OpenSAFELY platform. Lancet. 2021;397(10286):1711‐1724. 33939953 10.1016/S0140-6736(21)00634-6 PMC8087292

[8] Goff LM . Ethnicity and type 2 diabetes in the UK. Diabet Med. 2019;36(8):927‐938. 30614072 10.1111/dme.13895

[9] Bornstein SR , Rubino F , Khunti K , et al. Practical recommendations for the management of diabetes in patients with COVID‐19. Lancet Diabetes Endocrinol. 2020;8(6):546‐550. 32334646 10.1016/S2213-8587(20)30152-2 PMC7180013

[10] Holman N , Knighton P , Kar P , et al. Risk factors for COVID‐19‐related mortality in people with type 1 and type 2 diabetes in England: a population‐based cohort study. Lancet Diabetes Endocrinol. 2020;8(10):823‐833. 32798471 10.1016/S2213-8587(20)30271-0 PMC7426091

[11] Abuhasira R , Ayalon‐Dangur I , Zaslavsky N , et al. A randomized clinical trial of Linagliptin vs. standard of Care in Patients Hospitalized with Diabetes and COVID‐19. Front Endocrinol (Lausanne). 2021;12:794382. 35002970 10.3389/fendo.2021.794382 PMC8727772

[12] Kosiborod MN , Esterline R , Furtado RHM , et al. Dapagliflozin in patients with cardiometabolic risk factors hospitalised with COVID‐19 (DARE‐19): a randomised, double‐blind, placebo‐controlled, phase 3 trial. Lancet Diabetes Endocrinol. 2021;9(9):586‐594. 34302745 10.1016/S2213-8587(21)00180-7 PMC8294807

[13] Stokel‐Walker C . What do we know about covid vaccines and preventing transmission? BMJ. 2022;376:o298. 35121611 10.1136/bmj.o298

[14] Xie Z , St Clair P , Goldman DP , Joyce G . Racial and ethnic disparities in medication adherence among privately insured patients in the United States. PLoS One. 2019;14(2):e0212117. 30763400 10.1371/journal.pone.0212117 PMC6375669

[15] Bettge K , Kahle M , Abd El Aziz MS , Meier JJ , Nauck MA . Occurrence of nausea, vomiting and diarrhoea reported as adverse events in clinical trials studying glucagon‐like peptide‐1 receptor agonists: a systematic analysis of published clinical trials. Diabetes Obes Metab. 2017;19(3):336‐347. 27860132 10.1111/dom.12824

[16] Htike ZZ , Zaccardi F , Papamargaritis D , Webb DR , Khunti K , Davies MJ . Efficacy and safety of glucagon‐like peptide‐1 receptor agonists in type 2 diabetes: a systematic review and mixed‐treatment comparison analysis. Diabetes Obes Metab. 2017;19(4):524‐536. 27981757 10.1111/dom.12849

[17] Hussein H , Zaccardi F , Khunti K , et al. Efficacy and tolerability of sodium‐glucose co‐transporter‐2 inhibitors and glucagon‐like peptide‐1 receptor agonists: a systematic review and network meta‐analysis. Diabetes Obes Metab. 2020;22(7):1035‐1046. 32077218 10.1111/dom.14008

[18] Consoli A , Formoso G . Do thiazolidinediones still have a role in treatment of type 2 diabetes mellitus? Diabetes Obes Metab. 2013;15(11):967‐977. 23522285 10.1111/dom.12101

[19] Zaccardi F , Webb DR , Htike ZZ , Youssef D , Khunti K , Davies MJ . Efficacy and safety of sodium‐glucose co‐transporter‐2 inhibitors in type 2 diabetes mellitus: systematic review and network meta‐analysis. Diabetes Obes Metab. 2016;18(8):783‐794. 27059700 10.1111/dom.12670

