# Supplementary material for: Ethnicity and risks of severe COVID‐19 outcomes associated with glucose‐lowering medications: A cohort study
Source: Diabetes Obes Metab. 2022 Sep 29:10.1111/dom.14872. Online ahead of print. doi: 10.1111/dom.14872 (PMC9538196; doi:10.1111/dom.14872)
Supplement: Supplementary file 1 — APPENDIX S1 Supporting Information [file DOM-9999-0-s001.pdf]

# SUPPLEMENTARY MATERIAL

## Ethnicity and risks of severe COVID-19 outcomes associated with glucose-lowering medications: a cohort study

## METHODS

### Data sources and study population

This study has been conducted and reported following the RECORD guidelines. We extracted data from the QResearch database (version 45), which includes primary care data of people registered across 1,321 general practices (18% of the English population) linked to hospitalisation data (Hospital Episode Statistics), mortality register (Office for National Statistics, NHS Digital), and SARS-CoV-2 testing data (national infectious disease surveillance system, Public Health England). The study period started from the date of the first confirmed SARS-CoV-2 infection in the UK (index date, 24/01/2020) until 31/10/2020. We included 9,828,099 adults aged 18 years or older at index date and with at least 12 months of continuous prior registration: among them, we identified 624,771 people with a recorded diagnosis of type 2 diabetes prior to or at the index date.

### Exposures

There were two primary exposures identified through primary care health records: glucose-lowering medications and self-assigned ethnicity. We defined medication use as at least one prescription in the 3-month window before the index date: alpha-glucosidase inhibitors; dipeptidyl peptidase 4 inhibitors; thiazolidinediones; glucagon-like peptide-1 agonists; meglitinides; sodium-glucose cotransporter-2 inhibitors; sulphonylureas; metformin; and insulin. Self-reported ethnicity was recorded any time before the index date and grouped as: White; South Asian (Indian, Bangladeshi, Pakistani); Black (Black African, Caribbean), Asian (Chinese, Other Asian); Other; and not recorded/unknown.

### Outcomes

There were two outcomes of interest: COVID-19-related death and COVID-19-related hospitalisation. COVID-19-related death was defined as either confirmed or suspected COVID-19 on the death certificate, or a death from any cause with a confirmed positive SARS-CoV-2 test in the preceding 28 days.[1] COVID-19 hospitalisation was defined as any admission with confirmed positive COVID-19 rt-PCR test in the last 14 days or with an ICD-10 diagnosis code (U07.1 or U07.2) as the main reason for admission.[2,3]

### Confounders

Based on previous evidence,[4-11] we extracted information at or before the index date (baseline) on the potential confounders, including demographic, clinical, and lifestyle factors, shown in **Table 1**.

### Statistical analyses

We summarised baseline characteristics as frequencies (%) or mean and standard deviation, overall and stratified by ethnicity. For each outcome, we used complete-case Royston-Parmar regressions to estimate ethnicity-specific hazard ratios comparing prescription vs no prescription of the medication of interest in a model including confounders (**Table 1**), the number of glucose-lowering medication(s) (regardless of the pharmacological class), and an interaction term between ethnicity and the glucose-lowering medication of interest. Statistical significance for interactions was tested with the likelihood ratio test. Analyses were conducted in Stata v.17.

**Figure S1:** Mortality and hospitalisation rates for COVID-19

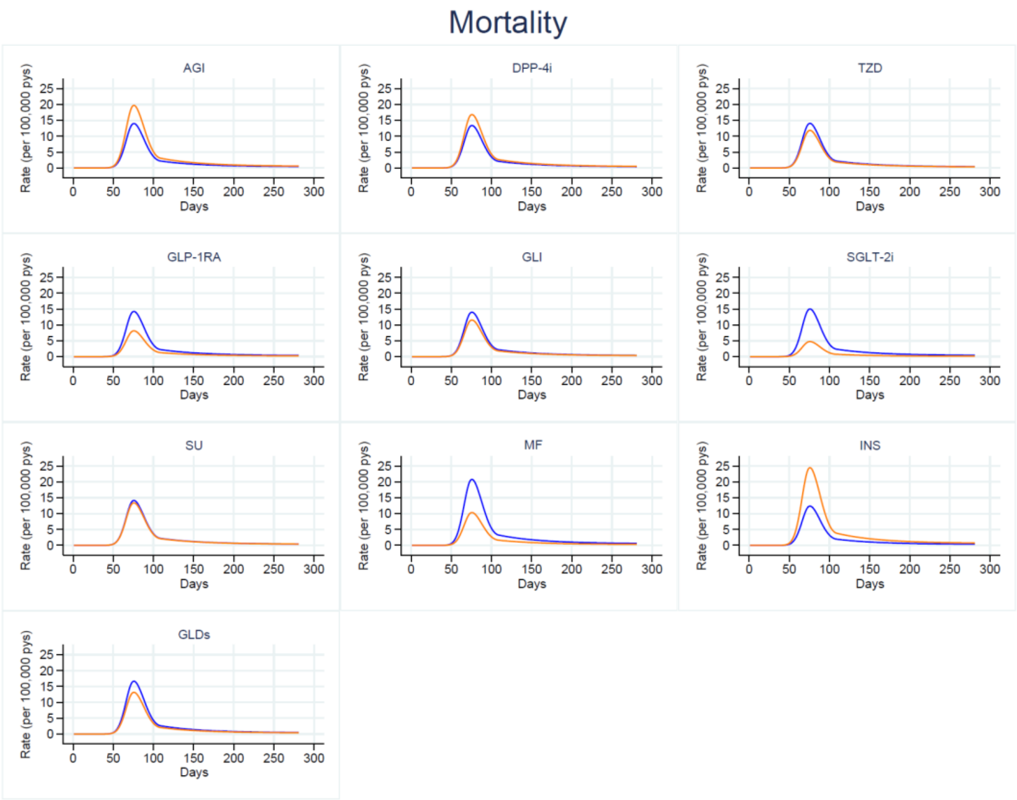


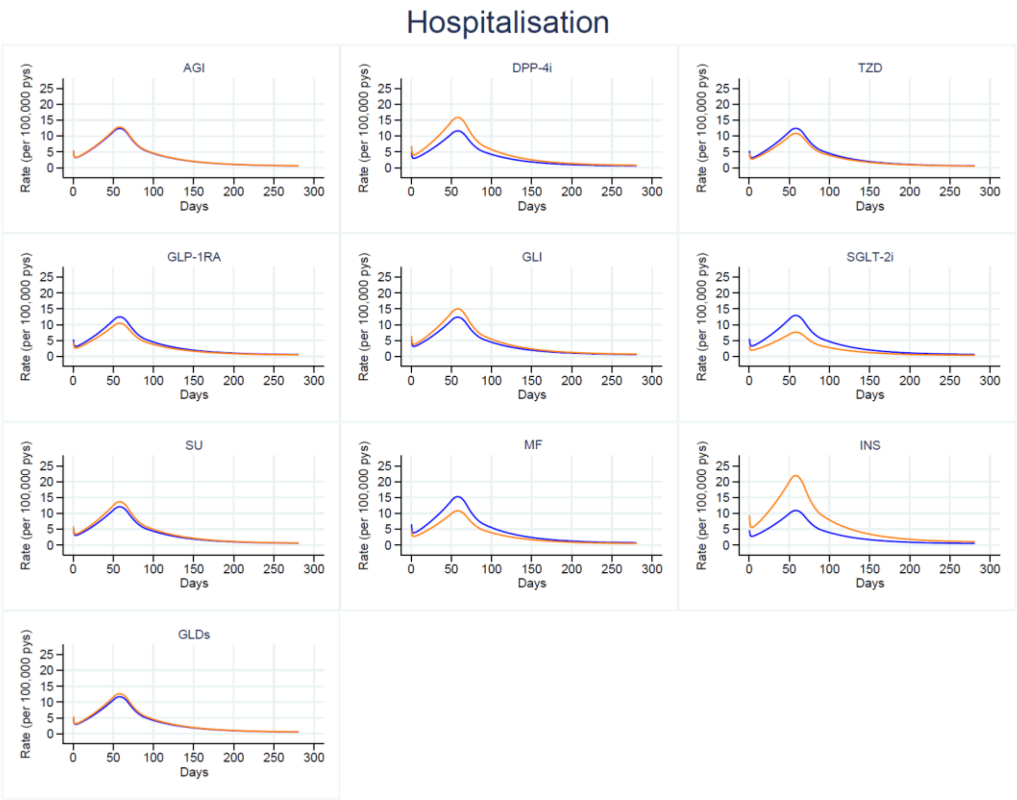


**AGI**: Alpha-glucosidase inhibitors; **DPP-4i**: Dipeptidyl peptidase 4 inhibitors; **TZD**: Thiazolidinediones; **GLP-1RA**: Glucagon-like peptide-1 agonists; **GLI**: Meglitinides; **SGLT-2i**: Sodium-glucose cotransporter-2 inhibitors; **SU**: Sulphonylureas; **MF**: Metformin; **INS**: Insulin; **GLDs**: Any glucose lowering medication.

Crude rates (per 100,000 person-years [pys]) of COVID-19 mortality and hospitalisation in people with (orange) vs without (blue) each glucose lowering medication; start of observation (index date): 24/1/2020.

## REFERENCES


1. GOV.UK. https://www.gov.uk/government/publications/phe-data-series-on-deaths-in-people-with-covid-19-technical-summary. Accessed on 31/8/2022.

2. GOV.UK. https://coronavirus.data.gov.uk/details/healthcare. Accessed on 31/8/2022.

3. Nyberg T, Twohig KA, Harris RJ, Seaman SR, Flannagan J, Allen H, et al. Risk of hospital admission for patients with SARS-CoV-2 variant B.1.1.7: cohort analysis. BMJ. 2021;373:n1412.

4. Singh AK, Gillies CL, Singh R, Singh A, Chudasama Y, Coles B, et al. Prevalence of co-morbidities and their association with mortality in patients with COVID-19: A systematic review and meta-analysis. Diabetes Obes Metab. 2020;22(10):1915-24.

5. Khunti K, Knighton P, Zaccardi F, Bakhai C, Barron E, Holman N, et al. Prescription of glucose-lowering therapies and risk of COVID-19 mortality in people with type 2 diabetes: a nationwide observational study in England. Lancet Diabetes Endocrinol. 2021;9(5):293-303.

6. Mathur R, Rentsch CT, Morton CE, Hulme WJ, Schultze A, MacKenna B, et al. Ethnic differences in SARS-CoV-2 infection and COVID-19-related hospitalisation, intensive care unit admission, and death in 17 million adults in England: an observational cohort study using the OpenSAFELY platform. The Lancet. 2021;397(10286):1711-24.

7. Hartmann-Boyce J, Rees K, Perring JC, Kerneis SA, Morris EM, Goyder C, et al. Risks of and From SARS-CoV-2 Infection and COVID-19 in People With Diabetes: A Systematic Review of Reviews. Diabetes Care. 2021;44(12):2790-811.

8. Wu J, Zhang J, Sun X, Wang L, Xu Y, Zhang Y, et al. Influence of diabetes mellitus on the severity and fatality of SARS-CoV-2 (COVID-19) infection. Diabetes Obes Metab. 2020;22(10):1907-14.

9. Nafilyan V, Islam N, Mathur R, Ayoubkhani D, Banerjee A, Glickman M, et al. Ethnic differences in COVID-19 mortality during the first two waves of the Coronavirus Pandemic: a nationwide cohort study of 29 million adults in England. Eur J Epidemiol. 2021;36(6):605-17.

10. Holman N, Knighton P, Kar P, O'Keefe J, Curley M, Weaver A, et al. Risk factors for COVID-19-related mortality in people with type 1 and type 2 diabetes in England: a population-based cohort study. Lancet Diabetes Endocrinol. 2020;8(10):823-33.

11. Bramante CT, Ingraham NE, Murray TA, Marmor S, Hovertsen S, Gronski J, et al. Metformin and risk of mortality in patients hospitalised with COVID-19: a retrospective cohort analysis. The Lancet Healthy Longevity. 2021;2(1):e34-e41.

## RECORD checklist

| | Item No. | STROBE items | Location in manuscript where items are reported | RECORD items | Location in manuscript where items are reported |
|---|---|---|---|---|---|
| **Title and abstract** | | | | | |
| | 1 | (a) Indicate the study's design with a commonly used term in the title or the abstract (b) Provide in the abstract an informative and balanced summary of what was done and what was found | Page 1 | RECORD 1.1: The type of data used should be specified in the title or abstract. When possible, the name of the databases used should be included.<br><br>RECORD 1.2: If applicable, the geographic region and timeframe within which the study took place should be reported in the title or abstract.<br><br>RECORD 1.3: If linkage between databases was conducted for the study, this should be clearly stated in the title or abstract. | Page 1; Abstract: NA |
| **Introduction** | | | | | |
| Background rationale | 2 | Explain the scientific background and rationale for the investigation being reported | Page 2 | | |
| Objectives | 3 | State specific objectives, including any prespecified hypotheses | Page 2 | | |
| **Methods** | | | | | |
| Study Design | 4 | Present key elements of study design early in the paper | Supplementary Material | | |
| Setting | 5 | Describe the setting, locations, and relevant dates, including periods of recruitment, exposure, follow-up, and data collection | Supplementary Material | | |
| Participants | 6 | *(a) Cohort study* - Give the eligibility criteria, and the sources and methods of selection of participants. Describe methods of follow-up | Supplementary Material | RECORD 6.1: The methods of study population selection (such as codes or algorithms used to identify subjects) should be listed in detail. If this is not possible, an explanation should be provided.<br><br>RECORD 6.2: Any validation studies of the codes or algorithms used to select the population should be referenced. If validation was conducted for this study and not published elsewhere, detailed methods and results should be provided. | Supplementary Material |

| | | | | | |
|---|---|---|---|---|---|
| | | | | RECORD 6.3: If the study involved linkage of databases, consider use of a flow diagram or other graphical display to demonstrate the data linkage process, including the number of individuals with linked data at each stage. | |
| Variables | 7 | Clearly define all outcomes, exposures, predictors, potential confounders, and effect modifiers. Give diagnostic criteria, if applicable. | Supplementary Material | RECORD 7.1: A complete list of codes and algorithms used to classify exposures, outcomes, confounders, and effect modifiers should be provided. If these cannot be reported, an explanation should be provided. | Supplementary Material |
| Data sources/ measurement | 8 | For each variable of interest, give sources of data and details of methods of assessment (measurement).<br>Describe comparability of assessment methods if there is more than one group | Supplementary Material | | |
| Bias | 9 | Describe any efforts to address potential sources of bias | Supplementary Material | | |
| Study size | 10 | Explain how the study size was arrived at | Supplementary Material | | |
| Quantitative variables | 11 | Explain how quantitative variables were handled in the analyses. If applicable, describe which groupings were chosen, and why | Supplementary Material | | |
| Statistical methods | 12 | (a) Describe all statistical methods, including those used to control for confounding<br>(b) Describe any methods used to examine subgroups and interactions<br>(c) Explain how missing data were addressed<br>(d) *Cohort study* - If applicable, explain how loss to follow-up was addressed<br>(e) Describe any sensitivity analyses | Supplementary Material | | |
| Data access and cleaning methods | | .. | | RECORD 12.1: Authors should describe the extent to which the investigators had access to the database population used to create the study population.<br><br>RECORD 12.2: Authors should provide information on the data cleaning methods used in the study. | Supplementary Material |
| Linkage | | | | RECORD 12.3: State whether the study included person-level, institutional-level, or other data linkage across two or more databases. The methods of linkage and methods of linkage quality evaluation should be provided. | Supplementary Material |

| | | | | | |
|---|---|---|---|---|---|
| **Results** | | | | | |
| Participants | 13 | (a) Report the numbers of individuals at each stage of the study (*e.g.*, numbers potentially eligible, examined for eligibility, confirmed eligible, included in the study, completing follow-up, and analysed)<br>(b) Give reasons for non-participation at each stage.<br>(c) Consider use of a flow diagram | Supplementary Material; page 3 | RECORD 13.1: Describe in detail the selection of the persons included in the study (*i.e.,* study population selection) including filtering based on data quality, data availability and linkage. The selection of included persons can be described in the text and/or by means of the study flow diagram. | Supplementary Material |
| Descriptive data | 14 | (a) Give characteristics of study participants (*e.g.*, demographic, clinical, social) and information on exposures and potential confounders<br>(b) Indicate the number of participants with missing data for each variable of interest<br>(c) *Cohort study* - summarise follow-up time (*e.g.*, average and total amount) | Page 3, Table 1 | | |
| Outcome data | 15 | *Cohort study* - Report numbers of outcome events or summary measures over time | Page 3-4, Table 1 | | |
| Main results | 16 | (a) Give unadjusted estimates and, if applicable, confounder-adjusted estimates and their precision (e.g., 95% confidence interval). Make clear which confounders were adjusted for and why they were included<br>(b) Report category boundaries when continuous variables were categorized<br>(c) If relevant, consider translating estimates of relative risk into absolute risk for a meaningful time period | Page 3-4, Table 1, Figure 1 | | |
| Other analyses | 17 | Report other analyses done—e.g., analyses of subgroups and interactions, and sensitivity analyses | Page 3, Figure S1 | | |
| **Discussion** | | | | | |
| Key results | 18 | Summarise key results with reference to study objectives | Page 5 | | |
| Limitations | 19 | Discuss limitations of the study, taking into account sources of potential bias or imprecision. Discuss both direction and magnitude of any potential bias | Page 5,6 | RECORD 19.1: Discuss the implications of using data that were not created or collected to answer the specific research question(s). Include discussion of misclassification bias, unmeasured confounding, missing data, and changing eligibility over time, as they pertain to the study being reported. | Page 5,6 |

| | | | | | |
|---|---|---|---|---|---|
| Interpretation | 20 | Give a cautious overall interpretation of results considering objectives, limitations, multiplicity of analyses, results from similar studies, and other relevant evidence | Pages 5,6 | | |
| Generalisability | 21 | Discuss the generalisability (external validity) of the study results | Pages 5,6 | | |
| **Other Information** | | | | | |
| Funding | 22 | Give the source of funding and the role of the funders for the present study and, if applicable, for the original study on which the present article is based | Page 7 | | |
| Accessibility of protocol, raw data, and programming code | | .. | Page 8 | RECORD 22.1: Authors should provide information on how to access any supplemental information such as the study protocol, raw data, or programming code. | Page 8 |

Pages refer to the original, word document submission.
